# COVID-19 Associated Myocarditis Clinical Outcomes among Hospitalized Patients in the United States: A Propensity Matched Analysis of National Inpatient Sample

**DOI:** 10.3390/v14122791

**Published:** 2022-12-14

**Authors:** Monique G. Davis, Aniesh Bobba, Prabal Chourasia, Karthik Gangu, Hina Shuja, Dima Dandachi, Asif Farooq, Sindhu Reddy Avula, Rahul Shekhar, Abu Baker Sheikh

**Affiliations:** 1 Department of Internal Medicine, University of New Mexico Health Sciences Center, Albuquerque, NM 87106, USA; 2 Department of Medicine, John H Stronger Hospital, Cook County, Chicago, IL 60612, USA; 3 Department of Hospital Medicine, Mary Washington Hospital, Fredericksburg, VA 22401, USA; 4 Department of Internal Medicine, University of Kansas Medical Center, Kansas City, KS 66160, USA; 5 Department of Medicine, Karachi Medical and Dental College, Karachi 74700, Pakistan; 6 Division of Infectious Diseases, University of Missouri-Columbia, Columbia, MO 65211, USA; 7 Department of Family and Community Medicine, Texas Tech Health Sciences Center, Lubbock, TX 79409, USA; 8 Department of Interventional Cardiology, Division of Cardiology, University of Kansas, St Francis Campus, Kansas City, KS 66606, USA

**Keywords:** COVID-19, myocarditis, mortality, prevalence, complications, United States, NIS

## Abstract

Coronavirus-19 (COVID-19), preliminarily a respiratory virus, can affect multiple organs, including the heart. Myocarditis is a well-known complication among COVID-19 infections, with limited large-scale studies evaluating outcomes associated with COVID-19-related Myocarditis. We used the National Inpatient Sample (NIS) database to compare COVID-19 patients with and without Myocarditis. A total of 1,659,040 patients were included in the study: COVID-19 with Myocarditis (*n* = 6,455, 0.4%) and COVID-19 without Myocarditis (*n* = 1,652,585, 99.6%). The primary outcome was in-hospital mortality. Secondary outcomes included mechanical ventilation, vasopressor use, sudden cardiac arrest, cardiogenic shock, acute kidney injury requiring hemodialysis, length of stay, health care utilization costs, and disposition. We conducted a secondary analysis with propensity matching to confirm results obtained by traditional multivariate analysis. COVID-19 patients with Myocarditis had significantly higher in-hospital mortality compared to COVID-19 patients without Myocarditis (30.5% vs. 13.1%, adjusted OR: 3 [95% CI 2.1–4.2], *p* < 0.001). This cohort also had significantly increased cardiogenic shock, acute kidney injury requiring hemodialysis, sudden cardiac death, required more mechanical ventilation and vasopressor support and higher hospitalization cost. Vaccination and more research for treatment strategies will be critical for reducing worse outcomes in patients with COVID-19-related Myocarditis.

## 1. Introduction

The Severe Acute Respiratory Virus Syndrome Coronavirus 2 (SARS-CoV-2) has caused worldwide pandemic affecting millions of people across the globe since it was first detected in Wuhan, China in December 2019 [1,2]. Though it is preliminarily a respiratory virus, it affects multiple organ systems including the cardiovascular system [3]. Cardiac complications can affect 20–30% of COVID-19 patients and lead to worse morbidity and mortality [4,5]. Cardiovascular complications can include myocarditis, acute myocardial infarction, heart failure, and arrhythmias [3]. Though myocarditis is associated with worse outcomes in COVID-19 patients [6], there are limited large-scale studies which evaluate outcomes associated with COVID-19-related myocarditis. 

Myocarditis association with COVID-19 infection is well established by multiple studies [6,7,8]. Myocarditis refers to inflammation of cardiac muscles caused by infectious and non-infectious etiologies [9,10,11]) with viral etiologies being most common in the United States and other developed countries [11]. It can present with focal or diffuse myocardial involvement and can be characterized as an acute, subacute, or chronic disease process [11,12]. Its symptoms and signs are highly variable and can include generalized fatigue, chest pain, sinus tachycardia, new onset congestive heart failure, arrhythmia, cardiogenic shock, and death [11,12,13]. It is frequently associated with elevated troponin, C-reactive protein, and erythrocyte sedimentation rate (ESR) [12]. Echocardiogram is part of the standard evaluation and can show reduced ejection fraction [12]. While endomyocardial biopsy is the gold standard for myocarditis diagnosis, Cardiac MRI is the gold standard for non-invasively assessing ventricular volumes, cardiac mass, and ejection fraction [12].

The pathophysiology of COVID-19-related myocarditis is hypothesized to involve a combination of immune-mediated damage and direct cytotoxic effects of the virus in myocardium [14]. Angiotensinogen-converting enzyme 2 (ACE2) receptor is found in multiple organ systems including cardiac tissue [14]. It is postulated to play a role in facilitating the entry of the virus into the cell by binding to the virus’s spike protein and causing direct cytotoxic effects [14]. 

Our study’s objective is to assess outcomes between COVID-19 patients with and without myocarditis utilizing data from National Inpatient Sample (NIS). The primary outcome was in-hospital mortality. Secondary outcomes included mechanical ventilation, vasopressor use, sudden cardiac arrest, cardiogenic shock, acute kidney injury (AKI) requiring hemodialysis (HD), length of stay (LOS), health care utilization costs, and disposition.

## 2. Materials and Methods

This retrospective study utilized NIS Healthcare Cost Utilization Project [HCUP] sponsored by the Agency for Healthcare and Research and Quality [AHRQ] database, which is an all-payer database that approximates a 20% stratified sample of discharges from US community hospitals [15]. In this analysis, we used the 2020 NIS data set, which included hospitalization from 1 January 2020 to 31 December 2020, and was made available to the public in October 2022. 

All patients 18 years of age and older admitted to the hospital with myocarditis and COVID-19 infection were included in this study. International classification of diseases 10th-clinical modification (ICD-10-CM) codes were used to retrieve patient samples with comorbid conditions, and ICD-10 procedure codes were used to identify inpatient procedures. A detailed code summary is provided in Table S1. Patients who were under the age of 18 years or were transferred out of the hospital were excluded from this study.

### 2.1. Covariates

The NIS database contains data regarding in-hospital outcomes, procedures, and other discharge-related information. Variables were divided into patient-related, hospital-related, and indicators of illness severity as below:

a. Patient: age, race, sex, comorbidities, insurance status, mean income in patient’s zip code, and disposition.
b. Hospital: location, teaching status, bed size, and region.
c. Illness severity: length of stay, mortality, hospitalization cost, Elixhauser comorbidity score [16], in-hospital complications, mechanical ventilation, circulatory support, and vasopressor use.

### 2.2. Study Outcomes

The primary outcome was in-hospital mortality. Secondary outcomes included mechanical ventilation, vasopressor use, sudden cardiac arrest, cardiogenic shock, acute kidney injury requiring hemodialysis, length of stay, health care utilization costs, and disposition.

### 2.3. Statistical Methods

Descriptive statistics were used to summarize the continuous and categorical variables. Continuous variables were summarized as mean ± SD; categorical data as the number and percentage. Univariate analyses for between-group comparisons used the Rao-Scott Chi-square test for categorical variables (e.g., sex and risk factors) and weighted simple linear regression for continuous variables (e.g., age). On an unmatched sample, univariate regression was used to identify independent variables (*p* ≤ 0.2), which were utilized to build a multivariate regression model. As our control group (COVID-positive without myocarditis) had a significantly higher sample than the test group (COVID-positive with myocarditis), we conducted a secondary analysis with propensity matching (PSM) to confirm results obtained by traditional multivariate analysis. Baseline demographics (age, race, sex, income status, insurance status) were matched using 1:1 nearest neighbor propensity score with 0.05 caliper width. On matched cohorts, a secondary multivariate regression model was built as described above. All analysis was performed using Stata 90 software version 17.0 (Stata Corporation, College Station, TX, USA). *p* values of less than 0.05 were considered statistically significant.

## 3. Results

### 3.1. Demographics and Baseline Comorbidities

**Figure 1:**
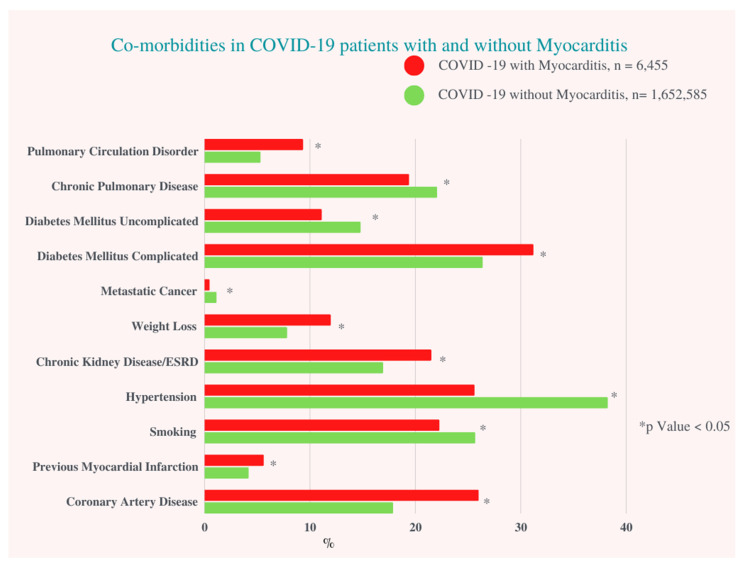
Co-morbidities in COVID-19 Patients with and without Myocarditis. ESRD: End Stage Renal Artery Disease.

In our study population, a total of 1,659,040 hospitalized COVID-19 patients between 1 January and 31 December 2020, and 6,455 (0.4%) patients diagnosed with COVID-19 infection with concomitant myocarditis were included. COVID-19 infection with myocarditis was more prevalent in males (61.4% vs. 52.0%, *p* < 0.001), had a greater proportion of Hispanics (23.2% vs. 21.5%, *p* = 0.02), Asians (4.2% vs. 3.2%, *p* = 0.02), African Americans (20.8% vs. 19.1%, *p* = 0.02), and Native Americans (1.4% vs. 1.0%, *p* = 0.02), and were more likely to have a household income above $80,000 (19.0% vs. 16.5%, *p* < 0.005) when compared to COVID-19 patients without myocarditis (Figure 1) (Table 1). 

Patients with COVID-19-associated myocarditis had more complicated diabetes mellitus (DM) (31.1% vs. 26.3%, *p* < 0.001), chronic kidney disease (CKD)/end stage renal disease (ESRD) (21.5% vs. 16.9%, *p* < 0.001), underlying coronary artery disease (CAD) (26% vs. 17.9%, *p* < 0.001) and history of past myocardial infarction (MI) (5.6% vs. 4.2%, *p* = 0.01). There was no significant difference regarding smoking status, history of previous percutaneous coronary intervention (PCI), alcohol use, or obesity between the two groups (Table S2). 

There was some variability in the geographic distribution of the patients and most patients with COVID-19 infection with and without myocarditis were seen at urban teaching hospitals (77.5% and 71.5%, respectively, *p* < 0.001). Moreover, both cohorts had a higher proportion of Medicare beneficiaries (53.5% vs. 53.2%, *p* = 0.466). Table 1 outlines the baseline characteristics of the study cohort.

### 3.2. In-Hospital Mortality

**Figure 2:**
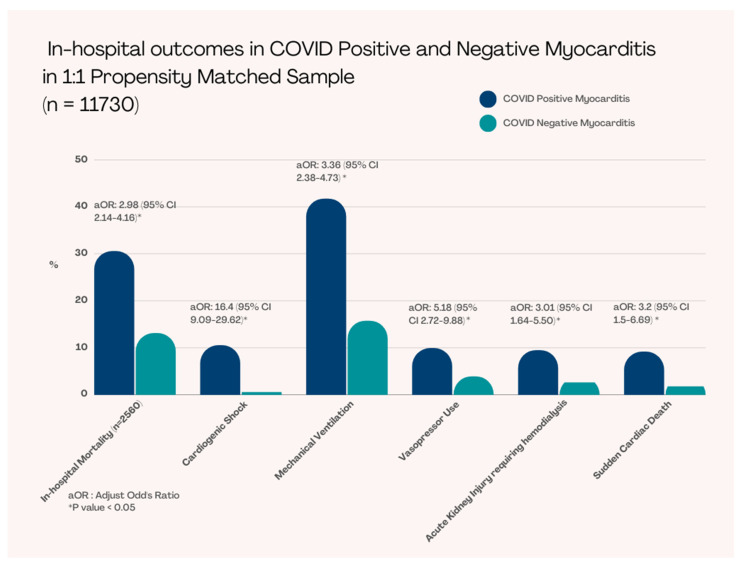
In-hospital Outcomes in COVID-19 Positive and Negative Myocarditis in 1:1 Propensity Matched Sample.

Propensity matching was performed regarding patient age, sex, race, income, and insurance status (Table 2). After PSM, we had a total of 5,864 patients in each group (with myocarditis and without myocarditis) (Table 2). The in-hospital mortality was significantly higher in COVID-19 patients with myocarditis compared to without myocarditis COVID-19 patients (30.5% vs 13.1%, adjusted OR [aOR]: 3 [95% CI 2.1–4.2], *p* < 0.001) (Figure 2) (Table 3).

**Figure 3:**
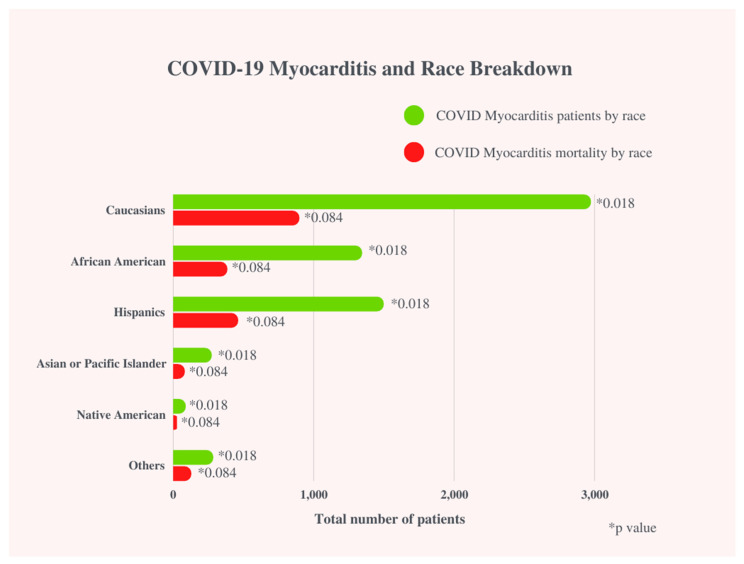
COVID-19 Myocarditis and Race Breakdown.

Mortality was further divided, evaluating gender, race, and age. Mortality in COVID-19 patients with myocarditis was significantly higher among younger patients: years ≥ 18–29 (1.5% vs. 0.6%, *p* < 0.001), years 30–49 (7.3% vs. 4.9%, *p* < 0.001, years 50–69 (39.1% vs. 30.4%, *p* < 0.001) as compared to COVID-19 patients without myocarditis. Mortality for patients with COVID-19 without myocarditis was significantly higher among older age patients: years ≥ 70 (64.1% vs. 52.0%, *p* < 0.001) when compared to COVID-19 patients with myocarditis. (Figure 3)

### 3.3. Mortality Predictors in COVID Myocarditis

**Figure 4:**
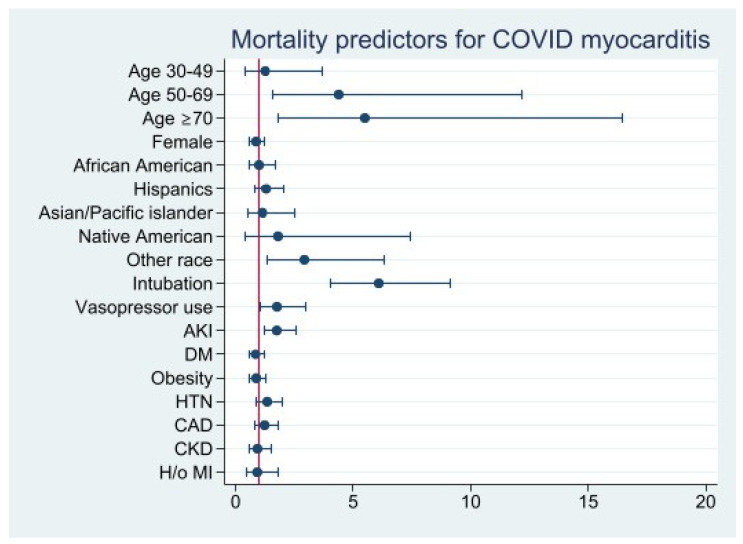
Mortality Predictors for COVID 19 Myocarditis.

Patients over the age of 50 had a significantly increased risk of mortality (age 50–69, aOR: 4.4 [95% CI 1.6–12.2], *p* = 0.005; age ≥ 70, aOR: 5.5 [95% CI 1.8–16.5], *p* = 0.002). Higher mortality was observed in patients requiring mechanical ventilation (aOR: 6.1 [95% CI 4.1–9.1], *p* < 0.001), vasopressor use (aOR: 1.8 [95% CI 1.0-3.0], *p* = 0.03), and developing AKI (aOR: 1.7 [95% CI 1.2–2.6], *p* = 0.004). Underlying cardiovascular comorbidities (i.e., CAD, hypertension, DM, CKD, history of previous MI, and obesity) were not statistically significant predictors of mortality in COVID-19 myocarditis patients (Figure 4) (Table S3).

### 3.4. In-Hospital Complications

Patients who presented with COVID-19 with myocarditis required more mechanical ventilation (41.7% vs. 15.8%, aOR: 3.4 [95% CI 2.4–4.7], *p* < 0.001), higher vasopressors use (9.9% vs. 3.9%, aOR: 5.2 [95% 2.7–9.9], *p* < 0.001), and experienced more cardiogenic shock (10.5% vs. 0.6%, aOR: 16 [95% CI 9.1–29.6], *p* < 0.001); however, did not require significant mechanical circulatory support (2.6% vs. 0.5%, aOR: 2.5 [95% CI 0.5–11.4], *p* = 0.29). These patients also had statistically significant higher rates of acute kidney injury requiring hemodialysis (9.5% vs. 2.6%, aOR: 3.0 [95% CI 1.6–5.5], *p* < 0.001) and sudden cardiac arrest (9.1% vs. 1.8%, aOR: 3.2 [95% CI 1.5–6.7], *p* = 0.002) (Table 3).

### 3.5. In-Hospital Quality Measures and Disposition

Patients with COVID-19 and myocarditis had an increased mean length of stay (11.6 days vs. 8.4 days, adjusted length of stay of 1.9 days, *p* = 0.007) which was not significantly higher than COVID-19 patients without myocarditis. Patients with COVID-19 and myocarditis had higher mean total hospitalization cost (173,226 USD vs. 72,072 USD, adjusted total cost 61,153 USD, *p* < 0.001). Of those patients who survived, no significant difference was noticed in the disposition from the hospital. Patients in the COVID-19 with myocarditis cohort were able to be discharged to home (51.2% vs. 51.6%, *p* = 0.01) and required skilled nursing or long-term acute care compared to the COVID-19 without myocarditis group (28.3% vs. 24.8%, *p* = 0.01) (Table 3).

## 4. Discussion

In this retrospective analysis, we identified 1,659,040 patients hospitalized between 1 January 2020 and 31 December 2020 with a diagnosis of COVID-19 infection, out of which 6455 (Prevalence–0.4%) were diagnosed with COVID-19 infection and myocarditis. Our cohort included 1,652,585 COVID-19 patients without myocarditis during this same study duration. Major findings of this study include (1) COVID-19 patients with myocarditis had significantly higher in-hospital mortality compared to COVID-19 patients without myocarditis; (2) Patients in the COVID-19 and myocarditis cohort had significantly increased cardiogenic shock, sudden cardiac death, acute kidney injury requiring hemodialysis, and required more mechanical ventilation and vasopressor support; (3) In-hospital mortality was higher among younger age patients with COVID-19 with myocarditis and older age (≥70 years) patients with COVID-19 without myocarditis; (4) Underlying cardiovascular comorbidities in COVID-19 patients with myocarditis were not predictors of increased mortality.

The prevalence of COVID-19-related myocarditis was between 0.1% to 0.4% in prior literature, which was similar to our prevalence data of 0.4% [6,7,8]. To our knowledge, our study is the largest study comparing outcomes in hospitalized patients with COVID-19 infection with and without myocarditis. 

In Boehmer et al. and Sawalha et al., patients with COVID-19-related myocarditis were predominantly males (59.3% and 58%, respectively) [7,17]. These studies were consistent with our study findings. This increased incidence in males can be explained as men are more likely to undergo cardiac remodeling, leading to development of myocardial fibrosis compared to women [18]. This is a result of testosterone and its pro-inflammatory effect, which increases myocardial inflammation, whereas estrogen (17-beta-estradiol) is protective and decreases inflammation [19]. Although in our study males are more predisposed to having COVID-19 and myocarditis than females, we found that gender was not a predictor of mortality.

In Ammirati et al., 7.8% of Hispanics and 15.7% of African Americans were affected COVID-19-associated myocarditis patients [12]. Our study suggests Hispanics, Asians, African Americans, and Native Americans are more likely to have COVID-19-related myocarditis but there was no significant difference in mortality when compared to COVID-19 without myocarditis patients. We did not find any large adult studies investigating race as a risk factor for mortality in COVID-19-induced myocarditis. Further studies are necessary to assess race as a risk factor for mortality in COVID-19-related myocarditis in adult patients. The disparities in these groups across various studies can be explained by socio-economic factors (i.e., medical debt and distrust, lack of access to appropriate healthcare, differences in care from white counterparts, and language barriers) that already predispose these populations to disadvantages [20,21]. 

In our study, patients with COVID-19 and myocarditis had significantly higher in-hospital mortality at 30.5%, which was similar to mortality of 27% to 30.0% reported by Ho et al. and Rubens et al. [6,22]. The association between myocarditis and mortality was evident after propensity matching of the groups. This high mortality can partly be the result of lack of effective and available COVID-19 therapies and no available vaccinations during the early pandemic. When mortality breakdown was performed, which analyzed gender, race, and age, it was found that although gender and race are significant factors in terms of contracting COVID-19-related myocarditis, these factors do not contribute significance toward in-hospital mortality, as discussed above. This differs from age; it was found that mortality was significant among younger age patients (under 70 years) within the COVID-19 and myocarditis cohort in comparison with COVID-19 without myocarditis. This was consistent with a reported age range of 45–70 and an average age of 52.3 years for patients with mortality from myocarditis in the literature [23,24]. Younger patients are more predisposed to mortality due to a robust immune system, leading to a cytokine and inflammatory storm and resulting in complications like that of myocarditis [23]. Moreover, mortality was significant in older age patients (≥70 years) within the non-myocarditis COVID-19 cohort, which correlated with the literature [25]. In Kang et al., elderly patients 70 years and older with COVID-19 infection had mortality of 10.9%–26.6% (varied based on geography) and was believed to be a result of aging immunity, increasing number of comorbidities, and impact of congregate housing [25]. 

Per our data, patients with COVID-19-associated Myocarditis were more likely to have complicated Diabetes mellitus, Chronic kidney disease/End stage renal disease, and have a history of coronary artery disease or myocardial infarction; however, having these underlying cardiovascular comorbidities was not determined to be a predictor of mortality in COVID-19 myocarditis. A multicenter case series by Laganà et al. noted a higher prevalence of cardiac diseases and risk factors in their COVID-19 myocarditis cohort but did not find significance in how it related to mortality and complications [26]. COVID-19-related myocarditis mortality is likely due to the burden of the inflammatory disease, independent of cardiovascular risk factors [27]. However, other studies suggest the opposite in that even the presence of one comorbidity increased the risk of mortality [6,7,8,28]. Prospective studies would be beneficial for clarification. Underlying mechanisms related to an increased rate of acute kidney injury in patients with myocarditis are unclear. Still, they may be related to decreased perfusion in the setting of new onset congestive heart failure, vasopressor use and shock [29,30].

Our cohort of COVID-19 patients with myocarditis had significantly increased cardiogenic shock, mechanical ventilation and vasopressor requirements, acute kidney injury requiring hemodialysis, and sudden cardiac death compared to patients with COVID-19 without myocarditis. This was consistent with a meta-analysis of 2,389 patients by Santoso et al., which suggested similar outcomes in that cardiac injury was associated with higher mortality (65%, RR: 8.0 [95% CI 5.1–12.3], *p* < 0.001), higher need for intensive care unit (ICU)-level care (79%, RR: 7.9 [95% CI 1.5–41.8], *p* = 0.01), and severe COVID-19 disease (RR: 13.8 [95% CI 5.5–34.5], *p* < 0.001) [31]. That study indicated severity of myocarditis correlated with troponin elevation and inflammatory markers and thus, explains why these patients have worse outcomes than non-myocarditis COVID-19 patients. Additionally, reliance on cardiac enzymes, electrocardiograms (EKGs), and clinical suspicion (irrespective of cardiac history) when echocardiograms and Cardiac MRIs are not available for confirmatory diagnosis or when use of these modalities was delayed due to the early pandemic isolation effect can explain poor outcomes, too [22,28,32]. One of the limitations of our data is it did not include lab values and imaging since NIS diagnoses are based on ICD-10 codes. History, exam, and laboratory data pertaining to myocarditis can be nonspecific and thus, recognizing myocarditis and treating patients early and aggressively are pertinent to improving morbidity and mortality [33]. 

Patients with co-diagnosis of COVID-19 and myocarditis in our study had significantly higher mean total hospitalization cost and longer length of stay than non-myocarditis COVID-19 patients. This is not surprising given patients with COVID-19 and myocarditis required higher levels of care, including stays in ICUs and life support measures, as discussed above. 

Management of COVID-19-related myocarditis is primarily supportive while treating the underlying COVID-19 infection. Steroids, antivirals, and comfort medications are typically given to COVID-19 patients with benefit but it remains unclear if these measures have any benefit directed toward myocarditis specifically [34,35,36]. Per Taggarsi, further insight is needed regarding the effect of remdesivir specifically on mitigating adverse cardiovascular events [37]. Further management can include treatment of complications and tertiary comorbidities that develop from the secondary myocarditis, such as heart failure, which should be treated based on current standards of care with goal-directed medical therapy (GDMT) [38,39]. 

In regard to vaccination against COVID-19 with m-RNA vaccines, there have been concerns attributed to myocarditis as a potential side effect. In a review article by Bozkurt et al., 61 cases of myocarditis were reported out of >300 million administered vaccines (< 0.00002%) [40]. In another review article by Verma et al., a direct causal relationship between vaccination and myocarditis could not be definitively established due to lack of tissue testing for antibodies [41]. Moreover, a study by Patone et al. noted that risk of extra myocarditis events in the month after COVID-19 vaccination to be significantly lower compared to risk of myocarditis after COVID-19 infection (1–10 events per million persons vs. 40 extra events per million) [42]. Based on these studies, the benefits of vaccination (i.e., decreased severity of COVID-19 disease, hospitalization, mortality, and risk of myocarditis) outweigh risks associated with vaccination [43]. As suggested by Bozkurt et al., a collaborative registry of myocarditis secondary to COVID-19 vaccination and secondary to COVID-19 disease with collected data on patient demographics, clinical presentation, and biomarkers (troponin, EKG, echocardiogram, Cardiac MRI) in conjunction with a paired registry of blood and cardiac tissue samples would be of value to answer many of these remaining questions [40]. 

Immune responses, including by pathogenic T-cells and inflammatory monocytes, may play a significant role in tissue damage (including myocardium) seen in COVID-19 infection [44,45]. Multi-omic profiling is an emerging approach to understanding immune responses associated with COVID-19 infection and disease severity. Su et al. identified a significant shift between mild and moderate disease in inflammatory signalling in plasma multi-omic analysis of 139 COVID-19 patients studied before the introduction of vaccination [46]. Increased inflammatory signals reflecting stress environments were noted with loss in metabolic resources and a drop in xenobiotic metabolism and postulated to influence the immune response in COVID-19 patients [46]. Sue et al. noted that disease severity is associated with non-monotonic evolution of CD8+ cell phenotypic composition (with increase in naive clusters and decrease in activated effector T cells noted in severe disease), non-monotonic changes of CD8+ T cells’ polyfunctionality and dysfunctional monocyte subpopulation. They also noted the transition from mild to moderate disease was accompanied by the expansion of CD8+ clones and two distinct CD4+ T cell phenotypes. These results were further supported by a study by Filblin et al., who studied the association of plasma proteomics with the role of immune cells and disease severity in 306 COVID-19 patients and 78 symptomatic controls over time [45]. They found an association between severe COVID-19 disease with heterogeneous plasma proteomic response. This study suggested the predominant role of viral infection and pre-existing comorbidities with advanced age rather than immune-mediated process in the underlying pathology of COVID-19 infection. Significant expansion of naive B cell and antibody-secreting cells in moderate to severe COVID-19 disease noted in this study may play a role in dysregulated humoral immunity response [45]. This study provided further evidence that plasma proteomes from circulating immune cells ( monocytes, plasmablasts, CD8+ T and NK cells) can play a direct role in tissue damage in multiple organs, including the heart [45]. Lee et al. in study of 198 COVID-19 patients with single cell multi-nomics and analysis of plasma metabolite found that increasing disease severity correlated with division of monocytes into two metabolically and functionally distinct subsets, metabolically hyperactive CD8+ T cell subpopulation, marked metabolic heterogeneity with CD4+ cells and metabolically dominant NK cell subpopulation [47]. This study further suggested metabolic reprogramming of immune cells with COVID-19 infection. These studies show that multi-omic profiling can be very promising to help undercover molecular underpinnings of myocarditis in COVID-19 patients. We were unable to perform multi-omic profiling due to limitations of the data obtained from the National Inpatient sample database. Future studies which evaluate multi-omic profiling of patients with COVID-19 and myocarditis will be very valuable to understand the pathophysiology of this association. 

COVID-19 has affected millions of people across the globe. Even though effective vaccination and treatment strategies have helped to reduce acute COVID-19 severity, its long-term effects four weeks after acute infection in the form of long COVID are increasingly being recognized [48]. In the study done by Puntmann et al. in patients with mild COVID infection, upto 57% of participants continued to have persistent cardiac symptoms with a median follow-up of 329 days (IQR 274–383 days) [49]. Myocarditis as part of long COVID has been reported in multiple studies [49,50,51,52,53]. In a large study by Patone et al. with people 16 years and older, an extra 40 myocarditis events per million were noted 1–28 days after a positive SARS-COV-2 test [42], while another study indicated that males aged 12–17 were likely to have 450 cases per million infections within three months of COVID-19 infection [52]. Mechanisms underlying potential long COVID and myocarditis need to be better understood. Su et al. performed a deep multi-omic longitudinal study to evaluate PASC (post-acute sequelae of COVID-19) anticipating biological factors [46]. One of the limitations of our study based on the 2020 NIS dataset is that we could not study long COVID given the ICD-10 code for long COVID was introduced in 2021 [54]. Prospective studies utilizing multi-omic analysis can enhance understanding of relationship and pathophysiology of long COVID and myocarditis. 

The NIS database has provided new information that can be used for the awareness of COVID-19-related myocarditis. It highlights the importance of stricter inpatient monitoring and cardiology outpatient follow-up for patients who develop cardiac complications. With this data, there can be further investigations into development of directed therapies for COVID-19-related myocarditis in order to improve mortality and the aforementioned outcomes. This data also allows for the promotion and emphasis of vaccinations. 

## 5. Limitations

Our study data was collected from the NIS which may introduce selection bias. Most patients with COVID-19 and myocarditis were diagnosed at urban teaching hospitals which have more resources like MRI compared to smaller hospitals. It may result in less final diagnosis of myocarditis at smaller hospitals despite the initial clinical suspicion. Diagnosis of myocarditis was based on ICD-10 codes as NIS data lacks lab values and imaging and may make it prone to errors. Our larger sample size helps to reduce the effects of these errors. The study cohort also included primarily non-vaccinated individuals as the FDA first approved COVID vaccination under EUA on 11 December 2020. It is possible COVID-19 vaccination may alter outcome in myocarditis between vaccinated and unvaccinated individuals.

## 6. Conclusions

Our study found that myocarditis in COVID-19 patients was associated with worse outcomes both in terms of morbidity and mortality, including increased acute kidney injury needing hemodialysis, cardiogenic shock with vasopressor support, mechanical ventilation and sudden cardiac death. Vaccination may play a pivotal role in reducing the incidence of COVID-19-related myocarditis. Increased risk of worse outcomes coupled with limited treatment options highlights the need for further research for effective vaccination and treatment strategies to improve outcomes in COVID-19-positive patients.

## Figures and Tables

**Table 1:** COVID-19 and Myocarditis Unmatched Patient-level Characteristics.

Characteristics	COVID 19 without Myocarditis	COVID 19 with Myocarditis	*p* Value
N = 1,659,040	N = 1,652,585 (99.6%)	N = 6455 (0.38%)	
Sex (Female)	47.98%	38.57%	<0.001
Mean Age Years (SD)			0.458
Male	63.43 (16.28)	61.16 (18.27)	
Female	63.06 (18.85)	65.57 (17.02)	
Age Groups			0.150
≥18–29	4.94%	6.12%	
30–49	16.8%	16.27%	
50–69	37.24%	35.4%	
≥70	41.02%	42.22%	
*Race			0.018
Caucasians	50.93%	46.06%	
African American	19.05%	20.8%	
Hispanics	21.46%	23.19%	
Asian or Pacific Islander	3.24%	4.22%	
Native American	1.03%	1.35%	
Others	4.29%	4.38%	
Median Household Income			0.046
<49,999 $	34.13%	30.76%	
50,000–64,999 $	27.19%	28.15%	
65,000–85,999 $	22.16%	22.08%	
>86,000 $	16.52%	19.01%	
Insurance Status			0.466
Medicare	53.24%	53.46%	
Medicaid	15.16%	14.56%	
Private	27.64%	27.18%	
Self-pay	3.96%	4.8%	
Hospital Division			<0.001
New England	3.79%	5.03%	
Middle Atlantic	14.59%	19.44%	
East North Central	15.54%	15.18%	
West North Central	6.7%	7.67%	
South Atlantic	20.09%	16.73%	
East South Central	6.72%	6.35%	
West South Central	14.33%	11.54%	
Mountain	6.92%	5.73%	
Pacific	11.29%	12.32%	
Hospital Bedsize			0.001
Small	24.35%	19.13%	
Medium	28.98%	30.36%	
Large	46.67%	50.5%	
Hospital Teaching Status			0.000
Rural	9.81%	7.59%	
Urban Non-teaching	18.67%	14.87%	
Urban Teaching	71.52%	77.54%	

**Table 2:** COVID-19 Propensity 1:1 Matched patient-level Characteristics ^2^.

Characteristics	COVID 19 without Myocarditis	COVID with Myocarditis	*p* Value
N = 11730	N = 5865	N = 5865	
Sex (Female)	39.22%	39.39%	0.935
Mean Age Years (SD)	63.39 (12.12)	63.33 (12.19)	0.939
Age Groups			0.998
≥18–29	5.8%	5.88%	
30–49	15.52%	15.69%	
50–69	35.64%	35.29%	
≥70	43.05%	43.14%	
Race			0.996
Caucasians	46.8%	46.89%	
African American	20.55%	20.46%	
Hispanics	23.1%	22.85%	
Asian or Pacific Islander	4.35%	4.35%	
Native American	0.94%	1.19%	
Others	4.26%	4.26%	
Median Household Income			0.999
<49,999 $	31.12%	31.12%	
50,000–64,999 $	27.45%	27.45%	
65,000–85,999 $	22.17%	22.25%	
>86,000 $	19.27%	19.18%	
Insurance Status			0.99
Medicare	53.2%	53.28%	
Medicaid	14.15%	14.15%	
Private	27.88%	27.71%	
Self-pay	4.77%	4.86%	
Hospital Division			<0.001
New England	73.49%	5.12%	
Middle Atlantic	20.55%	20.29%	
East North Central	3.32%	15.94%	
West North Central	0.85%	7.25%	
South Atlantic	0.77%	16.2%	
East South Central	^1^	6.05%	
West South Central	0.34%	11.59%	
Mountain	0.43%	5.63%	
Pacific	0.17%	11.94%	
Hospital Bedsize			<0.001
Small	30.61%	19.01%	
Medium	33.84%	30.52%	
Large	35.55%	50.47%	
Hospital Teaching Status			<0.001
Rural	2.98%	6.82%	
Urban non-teaching	10.32%	15%	
Urban teaching	86.7%	78.18%	

^1^ sample too small to report. ^2^ 1:1 PS matched variables: Age, sex, race, income, and insurance status.

**Table 3:** In-hospital Outcomes for 1:1 PS Matched Sample.

Variable	Patients without Myocarditis	Patients with Myocarditis	*p* Value
In-Hospital Mortality (N = 2560)	13.13% Adjusted odds ratio ^1^: 2.98(95% CI 2.14–4.16)	30.52%	<0.001
Vasopressor Use	3.92% Adjusted odds ratio ^1^: 5.18 (95% CI 2.72–9.88)	9.89%	<0.001
Mechanical ventilation	15.77% Adjusted odds ratio ^1^: 3.36 (95% CI 2.38–4.73)	41.69%	<0.001
Acute kidney Injury requiring hemodialysis	2.64% Adjusted odds ratio ^1^: 3.01 (95% CI 1.64–5.50)	9.46%	<0.001
Sudden Cardiac Arrest	1.79% Adjusted odds ratio ^1^: 3.2 (95% CI 1.5–6.69)	9.12%	0.002
Cardiogenic Shock	0.6% Adjusted odds ratio ^1^: 16.4 (95% CI 9.09–29.62)	10.49%	<0.001
Mechanical Circulatory Support (LVAD ^2^ or pVAD ^3^ or ECMO ^4^)	0.51% Adjusted odds ratio ^1^: 2.34 (95% CI 0.48–11.4)	2.64%	0.292
Mean Total Hospitalization Charge ($)	72,072 $Adjusted total charge ^1^: 61,153 $ higher	173,226$	<0.001
Mean Length of Stay (Days)	8.4 Adjusted length of stay^1^: 1.9 day higher	11.7	0.007
Disposition			0.097
Home/Routine	51.58%	51.17%	
SNF/LTAC/Nursing home	24.82%	28.31%	
Home Health	22.08%	20%	
AMA	1.53%	0.52%	

^1^ Adjusted for discharge quarter, Elixhauser comorbidities, hospital location, teaching status and bed size; ^2^ LVAD: Left Ventricular Assist Device; ^3^ pVAD: percutaneous Ventricular Assist Device; ^4^ ECHO: Extracorporeal membrane oxygenation.

## Data Availability

Restrictions apply to the availability of these data. Data was obtained from the National Inpatient Sample database, US.

## Supplementary Materials

The following supporting information can be downloaded at: https://www.mdpi.com/article/10.3390/v14122791/s1, Table S1: ICD-10 Codes; Table S2: Baseline co-morbidities of unmatched sample; Table S3: Mortality predictors for COVID-19 Myocarditis
