# Supplementary material for: COVID-19 Associated Myocarditis Clinical Outcomes among Hospitalized Patients in the United States: A Propensity Matched Analysis of National Inpatient Sample
Source: Viruses. 2022 Dec 14;14(12):2791. doi: 10.3390/v14122791 (PMC9785561; doi:10.3390/v14122791)
Supplement: Supplementary file 1 [file viruses-14-02791-s001.zip › viruses-2092854-supplementary.pdf]

Supplementary Table S1

Diagnosis	ICD-10 code
Myocarditis	B3320, B3322, B3324, I40, I400, I401, I408, I409, I41, I514,
COVID 19	U071, U00, U49, U50, U85, J1282
Mechanical ventilation	5A1945Z,5A1955Z,5A1935Z,5A09357,5A09457,5A09557
Vasopressor	3E030XZ,3E033XZ,3E040XZ,3E043XZ,3E050XZ,3E053XZ,3E060XZ,3E063XZ
Sudden cardiac arrest	I46, I97
Acute kidney injury and hemodialysis	N17, N990,5A1D70Z,5A1D90Z,5A1D80Z,5A1D00Z,5A1D60Z
Cardiogenic Shock	R570
Mechanical Circulatory Support	5A02110, 5A02210, 5A0211D, 02HA3RZ, 5A02116, 5A0221D, 5A1522F, 5A1522G, 5A1522H, 5A15A2F, 5A15A2G, 5A15A2H, 5A15223
Chronic Kidney Disease	N181, N182, N1830, N1831, N1832, N184,N185,N189
Pulmonary Circulation Disorder	Data Obtained from elixhauser comorbidity index
Chronic Pulmonary Disease	
Diabetes Uncomplicated	
Diabetes Complicated	
Hypothyroidism	
Peptic Ulcer Disease (excluding bleeding)	
Lymphoma	
Metastatic Cancer	
Solid Tumor Without Metastasis	
Rheumatoid Arthritis/Collagen Vascular	
Obesity	
Drug Abuse	
Hypertension	
Peripheral arterial disease	
Obstructive sleep apnea	
Liver Disease	
Alcohol	
Smoking	F17, F172, F1720, F17200, F17201, F17203, F17208, F17209, F1721, F17210, F17211, F17213, F17218, F17219, F1722, F17220, F17221, F17223, F17228, F17229, F1729, F17290, F17291, F17293, F17298, F17299, Z87891
History of PCI*	Z986, Z9861, Z9862
History of CABG	Z951
Previous Myocardial Infarction	I252
Coronary Artery Disease	I2510, I25111, I25118, I25119, I252, I253, I254, I2541, I2542, I255, I256, I257, I2570, I25700, I25701, I25708, I25709, I2571, I25710, I25711, I25718, I25719, I2572, I25720, I25721, I25728, I25729, I2573, I25730, I25731, I25738, I25739, I2575, I25750, I25751, I25758, I25759, I2576, I25760, I25761, I25768, I25769, I2579, I25790, I25791, I25798, I25799, I258, I2581, I25810, I25811, I25812, I2582, I2583, I2584, I2589, I259

*PCI: Percutaneous Coronary Intervention

Supplementary Table S2

Comorbidities	COVID -19 without Myocarditis	COVID-19 with Myocarditis	p Value
Elixsum	3.7(SD 2.2)	4.7(SD 2.2)	0.000
Pulmonary Circulation Disorder	5.29%	9.3%	0.000
Chronic Pulmonary Disease	22.01%	19.36%	0.026
Diabetes, Uncomplicated	14.76%	11.08%	0.000
Diabetes, Complicated	26.34%	31.14%	0.000
Hypothyroidism	13.2%	12.39%	0.392
Peptic Ulcer Disease (excluding bleeding)	0.32%	.46%	0.347
Lymphoma	0.8%	0.62%	0.481
Metastatic Cancer	1.1%	0.46%	0.025
Solid Tumor Without Metastasis	2.4%	1.78%	0.110

Rheumatoid Arthritis/Collagen Vascular disease	2.92%	3.72%	0.121
Obesity	25.6%	26.65%	0.416
Weight Loss	7.8%	11.93%	0.000
Drug Abuse	2.58%	2.09%	0.255
CKD/ESRD[1]	16.9%	21.46%	0.000
Hypertension	38.18%	25.56%	0.000
Peripheral Arterial Disease	1.6%	1.7%)	0.848
Obstructive Sleep Apnea	8.1%	7.4%	0.338
Liver Disease	3.9%	4.57%	0.234
Alcohol	2.38%	3.1%	0.095
Smoking	25.62%	22.23%	0.005
History of PCI[2]	0.43%	0.39%	0.799
History of CABG[3]	3.48%	3.72%	0.634

Previous Myocardial Infarction	4.16%	5.58%	0.013
Coronary Artery Disease	17.85%	25.95%	0.000

[1]CKD/ESRD: Chronic Kidney Disease/End Stage Renal Artery Stenosis; [2]PCI: Percutaneous Coronary Intervention; [3]CABG: Coronary Artery Bypass Graft

Supplementary Table S3

Mortality predictors for COVID-19 Myocarditis

Variable	aOR	95% CI (LL-UL)	P Value
**Age**			
≥18-29	Comparison		
30-49	1.25	0.42-3.68	0.67
50-69	4.38	1.58-12.15	0.005
≥70	5.49	1.83-16.46	0.002
**Female**	0.86	0.59-1.66	0.40
**Race**			
White	Comparison		
African American	0.99	0.59-1.66	1
Hispanics	1.3	0.84-2.01	0.23

Asians/Pacific Islander	1.13	0.51-2.52	0.74
Native	1.79	0.43-7.43	0.42
Others	2.92	1.35-6.31	0.006
Mechanical ventilation	6.09	4.06-9.14	<0.001
Vasopressor use	1.76	1.04-2.99	0.03
Acute Kidney Injury	1.74	1.19-2.55	0.004
Hypertension	1.34	0.9-2.01	0.14
Diabetes Mellitus	0.84	0.59-1.2	0.35
Coronary Artery Disease	1.23	0.83-1.83	0.28
Chronic Kidney Disease	0.92	0.57-1.5	0.75
Old Myocardial Infarction	0.9	0.46-1.78	0.77
Obesity	0.88	0.59-1.3	0.52
